# Completion of the maternal continuum of care in Kenya and Tanzania: role of quality antenatal care and other factors in a cross-sectional study

**DOI:** 10.21203/rs.3.rs-8901007/v1

**Published:** 2026-03-20

**Authors:** James Orwa, Samwel Maina Gatimu, Lucy Nyagah, Hussein Kidanto, Marleen Temmerman

**Affiliations:** Aga Khan University; Diabetes Foot Foundation of Kenya; Aga Khan University; Aga Khan University; Aga Khan University

**Keywords:** continuum of care, maternal and newborn health, Skilled birth attendants, postnatal care, quality antenatal care

## Abstract

**Introduction:**

Despite global decline in the maternal mortality, the decline in sub-Saharan African is still stagnating compared to other regions. Kenya and Tanzania had progressed in reducing maternal mortalities through different interventions including investing in interventions to improve the coverage of continuum of care, however, there is limited region-specific information on the subject. The study aimed to assess the coverage and determinants of maternal continuum of care among women who delivered in the last two years before surveys conducted in regions of Kenya and Tanzania.

**Methods:**

The study used data from end line survey of AQCESS and IMPACT project conducted in Kenya and Tanzania, respectively between 2020 and 2021. Cluster sampling approach with the village and household considered as the highest and lowest levels respectively was used to select women who delivered two years before the surveys. Maternal continuum of care is defined as attending at least four antenatal care, skilled birth attendance and receiving postpartum check-up within 48 hours. Socio-economic and reproductive variables were collected. Data were explored descriptively to assess coverage and multivariable logistic regression with robust standard errors was used to identify factors associated with CoC.

**Results:**

A total of 305 and 1049 women were included in Kenya and Tanzania surveys, respectively. The median age in years was 27.0 (IQR: 23.0–33.0). Women who attended at least four antenatal care visits were 75.5%, whilst 87.8% and 62.2% had skilled birth attendants and received postnatal check-up within 48 hours after delivery, respectively. The overall CoC was 31.1% (95% CI:28.6%–33.6%), with 26.9% (95% CI:22.0%–32.2%) in Kenya and 32.3% (95% CI:29.5%–35.2%) in Tanzania. Women with at least secondary level of education were 78% likely to complete Coc (aOR: 1.78; 95%CI: 1.13–2.84) than those with primary level of education. Similarly, women who received high quality antenatal care had 50% chances of completing CoC. However, not involving a woman in decision making about her own health and delayed initiation of antenatal care visits were associated with reduced odds of completing CoC.

**Conclusion:**

There were less than 50% coverage of complete CoC in the settings because of low coverage of postpartum check-up within 48 hours after delivery which varied between the regions. Having at least secondary level of education and receiving high quality antenatal care were associated with increased odds of completing CoC. Investment in girls’ education and strengthening the existing health systems to improve quality of antenatal care, which is the entry point into preventive and curative care services should be promoted to improve access to postpartum check-up and increase the coverage of completing CoC.

## Introduction

Despite a decrease in global maternal mortality ratio (MMR) by more than a third between 2000 and 2020, 287,000 women died due to complications related to pregnancy and childbirth globally in 2020 [1]. Notably, 95% of these deaths occurred in low- and lower-middle-income countries [1]. Sub-Saharan Africa had the highest neonatal mortality rate in the world at 27 deaths per 1000 live births, followed by central and southern Asia, with a neonatal mortality rate (NMR) of 21 deaths per 1000 live births [2].

In the past decade, Kenya and Tanzania have reduced maternal and neonatal mortality. The MMR and NMR reduced from 448 to 342 per 100,000 live births [1, 3, 4] and 22 to 21 deaths per 1,000 live births between 2008 and 2022 in Kenya [5]. In Tanzania, the MMR and NMR reduced from 530 to 104 deaths per 100,000 live births and 25 to 19 deaths per 1,000 live births between 2016 and 2022 [6]. These gains have occurred alongside improvements in key service-delivery indicators across the maternal continuum of care. During the same period, key maternal care indicator of antenatal care, health facility delivery and postnatal check-up within two days improved. In Kenya and Tanzania, 97.9% and 89.7% received at least one antenatal care with 66.4% and 65.1% attending at least four ANC visits, 82.3% and 81.2% delivered at a health facility and 72.5% and 50.5% received postpartum services, respectively. These patterns suggest progressive strengthening of linked maternal health services, although gaps remain in ensuring that women complete the full continuum of care.

Maternal continuum of care is a framework promoted by the World Health Organization to improve timely use of high-quality reproductive, maternal, newborn, and child healthcare services delivery throughout the lifecycle from pre-conception, pregnancy, delivery, and the postpartum period and across different levels of health systems. Strengthening the continuum is crucial in preventing maternal and neonatal deaths [7]. Most of these deaths result from preventable causes occurring across the continuum from pregnancy to the immediate postpartum period. During pregnancy, conditions such as preeclampsia/eclampsia and maternal anemia increase risks for both mothers and babies and contribute to prematurity. During labor and delivery, complications including uterine rupture and other intrapartum-related events can be fatal without timely and quality obstetric care. In the immediate postpartum period, postpartum hemorrhage remains a leading cause of maternal death, while newborn deaths are commonly due to infections such as sepsis, tetanus, and pneumonia [8] [2, 9].

Our previous study examined maternal healthcare services in isolation in Tanzania using baseline survey data [10–12], highlighting the need for a more integrated approach across the continuum of care. This study therefore aims to assess the coverage and determinants of completion of the maternal continuum of care in selected regions of Kenya and Tanzania, with a specific focus on the pregnancy, delivery, and immediate postpartum periods. The findings are expected to inform the co-creation of context-relevant interventions to improve the utilization and continuity of maternal health services from pregnancy through the first 48 hours after birth and ultimately contribute to improved maternal health indicators.

## Methods

### Study design, populations, sampling and setting

The study utilized secondary data from the end-line surveys of two projects—Access to Quality Care through Extending and Strengthening Health Systems (AQCESS) and Improving Access to Reproductive, Maternal and Newborn Health (IMPACT)—implemented in Kilifi (Kaloleni and Rabai sub-counties) and Kisii (Bomachoge and Borabu sub-counties) counties in Kenya and Mwanza region of Tanzania (Illemela, Nyamagana, Buchosa, Sengerema, Ukerewe, and Magu districts), respectively. The Kenyan survey was conducted in November and December 2020 while the Tanzania survey was conducted in March 2021. The surveys used a two-stage cluster sampling approach: the first stage where all the first 30 villages were selected based on the number of households they had and the second stage involved a random selection of households within the selected clusters. There was no further sampling at the household level and all consenting women of reproductive age (15–49 years) were surveyed. Details on design, population, sampling and settings can be found in our previous publications [10, 13].

### Data collection and management

In both surveys, data were collected electronically using open data kits by trained research assistants fluent in English and other local languages (Swahili, Giriama, Sukuma, and Ukerewe). The data collected were reviewed by the team leaders at the end of the day’s work before uploading them into the cloud server for further analysis. The questionnaire was first designed in English and later back translated into different local languages before converted into electronic data tool. The major sections were on reproductive, maternal, newborn, child and adolescent health. All the selected households were included in the interviews; in case there was no eligible respondent available at the time of data collection, three attempts were made before the households were declared unavailable. The research assistants had the support of village elders and village executive offices in Kenya and Tanzania, respectively.

### Study variables

#### Dependent variable

The main outcome of interest was complete CoC of maternal care which was measured as a composite score of at least four antenatal care (ANC), skilled birth attendance (SBA) and maternal immediate postnatal care (PNC) within 48 hours after delivery. A woman was considered to have completed CoC if she had received at least four antenatal care visits (ANC4+), delivered the recent birth with the assistant of skilled health providers(SBA) and received PNC within 48 hours of delivery (immediate PNC), otherwise not complete if she did not receive at least ANC4+, SBA or PNC. The definition was consistent with prior maternal continuum-of-care studies that measure continuity across pregnancy, delivery, and the immediate postpartum transition [14, 15].

#### Independent variables

The selection of independent variables guided by previous studies on maternal CoC [16–22] and the availability of data from the end-line surveys. The sociodemographic variables included maternal age (15–19, 20–34 and 35–49), women’s education (no formal, primary and secondary and higher), marital status (married, not married), woman decision making for seeking healthcare (woman alone, woman with someone and not involved) and household wealth quintiles (richest, richer, middle, poorer and poorest). The household wealth index, a composite measure of the cumulative living standard of a household, was calculated using principal component analysis based on household characteristics and household assets. Maternal variables included pregnancy intention during the last pregnancy and timing of first ANC. Quality of antenatal care was generated as composite variable consisting of blood pressure measured, blood sample has taken, urine sample was taken, received at least two tetanus injection, iron supplementation, and received drugs for intestinal worms. This dichotomized in adequate (high quality) if the woman received all these services, otherwise the antenatal care was considered inadequate [23].

#### Statistical analysis

The data were pooled from the two surveys, and analyzed descriptively using frequency and percentages for categorical variables and median and inter-quartile range for continuous variables. Overall, country-specific and region-specific prevalence of CoC was calculated including the 95% confidence interval. The study followed the approach of Wenjuan et al. [24] and Gizachew et al. [25] to fit four sequential multivariable logistic regression analyses with robust standard errors to analyze the factors associated with at least 4 antenatal care (ANC4+)(Model I), ANC4+ and SBA (Model II), SBA+PNC (Model III) and CoC (Model IV). Each of the model had different outcomes as defined: Model I was coded 1 if a woman received at least four antenatal care and 0 otherwise. Model II outcome was coded 1 for women who received at least four antenatal care and skilled birth attendance, and 0 for receiving at least four antenatal care visits but not skilled birth attendance. The outcome for Model III was coded as 1 for receiving skilled birth attendance and immediate postnatal care, and 0 for receiving skilled birth attendance but not immediate postnatal care, and finally the outcome for Model IV was coded as 1 for receiving antenatal care, skilled birth attendance, and immediate maternal postnatal care, and 0 otherwise.

#### Ethical considerations

The surveys were approved by the Aga Khan University Hospital Institutional Scientific Ethics and Research Committee in both countries. The research permits were obtained from NACOSTI and COSTECH for the projects in Kenya and Tanzania respectively. Further approvals to conduct the study in these regions were obtained from county Ministry of Health in Kenya and regional medical officer in Mwanza. The studies were conducted according to the rules and regulations approved by the research committees and in line with the Helsinki Declaration on Research involving Human Subjects. All participants provide informed consent by signing or using thumb prints on the consent forms before the start of the data collection. Women between the ages 15–17 years provided assent in addition to their parents or guardian consents.

## Results

### Respondents’ characteristics

The study included a total of 1,354 women who delivered in the two regions before the survey which consisted of 305 and 1,049 from Kenya and Tanzania, respectively. The overall median age in years was 27.0 (IQR: 23.0–33.0) with no significant difference in median ages in the two countries (p-value=0.057). Majority of the women were aged 20–34 years in both countries with teenagers being the minority group. Similarly, majority of the women had primary level of education with higher proportion with primary level of education in Tanzania compared to Kenya. Overall, most women (n=472, 34.9%) were from household in the second wealth quintile, however, by regions, most Kenyan women were from households in the first wealth quintile whilst in Tanzania, majority were in the second wealth quintile (Table 1).

In both countries, women were not involved in decision making about their own health as reported by 161 (52.8%) and 425 (40.5%) of the women in Kenya and Tanzania, respectively. The median time to initial antenatal care in weeks in Kenya was higher at 20.0 (IQR:14.0–24.0) as compared to Tanzania at 16.0 (IQR: 12.0–20.0). This translates to 546 (40.8%) of the women initiating antenatal care during the first trimester, 548 (41.0%) during the second trimester and 243 (18.2%) in the third trimester. Higher proportion of women in Tanzania initiated their antenatal during the first trimester as compared to Kenya and only 14.8% (n=155) of them initiated their visits in the third trimesters. However, higher proportion of women reported high-quality antenatal care compared to Tanzania at 81.0% (n=247) and 647 (61.7%), respectively. Similarly, a slightly higher proportion of women in Kenya wanted the most recent pregnancy than women in Tanzania (Table 1).

### Maternal continuum of care

Overall, only 31.1% (95% CI:28.6%–33.6%) of women completed the maternal continuum of care, with 26.9% (95% CI:22.0%–32.2%) in Kenya and 32.3% (95% CI:29.5%–35.2%) in Tanzania. The highest completion rate was in Buchosa at 40.1%, and the lowest in Sengerema at 18.2% (Table 2).

Most women attended at least four ANC visits (75.7%), with the highest percentage in Ilemela, Tanzania (81.3%) and the lowest in Kilifi, Kenya (67.7%). Most women had skilled birth attendance (87.8%), with the highest percentage in Buchosa, Tanzania (96.8%) and the lowest in Kilifi, Kenya (72.9%). At least, 62.2% of women received postnatal care within two days after delivery, with the highest percentage in Ukerewe, Tanzania (87.8%) and the lowest in Sengerema, Tanzania (41.2%) (Table 2).

Kenyan women had higher rates of skilled births and postnatal check-ups compared to Tanzanian women, at 14.1% and 8.0%, respectively. Women with at least four antenatal visits and skilled births were 27.5% in Kenya and 39.2% in Tanzania. Those with both antenatal visits and postnatal check-ups were 0.7% in Kenya and 2.4% in Tanzania (Table 2).

### Determinants of completion of continuum of care

Women not involved, secondary+ level of education, second and third trimester and having received high-quality antenatal care were significantly associated with the maternal continuum of care (Model IV). Women who were not involved in decision-making about their health had a decreased likelihood of completing the continuum of care (aOR: 0.66, 95% CI: 0.47–0.92). Similarly, women who attended their first antenatal care visits during the second or third trimester were less likely to complete the continuum of care. However, there were increased odds of completing the continuum of care among women with at least secondary education (aOR: 1.78, 95% CI: 1.13–2.84) or who have received quality antenatal care services (aOR: 1.50, 95% CI: 1.14–1.97). Other factors not significantly associated at p-value > 0.05 with the maternal continuum of care is presented in Table 3.

## Discussion

The study aimed to determine the coverage and determinants of completion of maternal continuum of care in some regions of Kenya and Tanzania. The study revealed less than half of the women completed the maternal CoC similar pattern to a study from 32 countries in sub-Saharan Africa of 29.4% [26] and in Ethiopia that found maternal completion rate of 37% [27] but lower than completion rate among Ghanian women of 8.0% [17]. Greatest contributor to the low rate of maternal continuum of care was postnatal care within two days after delivery which was stricter as the definition used in other studies. The slight variations in the completion rates could further be associated with the different interventions used in different settings to improve the uptake of the different components of continuum of care.

The study found women who had at least secondary education had higher odds of complete continuum of care than those with no formal education training which is in line with other studies [28]. Similarly, women with at least primary level of education showed high likelihood of continuation of care with the skilled birth attendance after receiving at least four antenatal care which was similar to the study in Ethiopia [25]. Low literacy levels among participants in this setting may be associated with low health knowledge, on the otherhand, highly educated women may acquire information from many sources including in schools regarding benefits of maternal health care. In addition, illiterate women are most likely economically unstable and, in many cases, depends on their husbands to finance their maternal needs including financial support to attend adequate maternal health care during the continuum of care process. Economic stability had shown strong associations with the uptake of maternal healthcare services in other settings [29].

On the other hand, women who were not involved in decision making about their health were less likely to have a complete continuum of care as compared to those who participated in the decision-making about their own health. Involving a woman in each stage of the continuum ensure improves maternal nutrition, and better maternal care, and provides a woman with the freedom to choose when and where to seek maternal healthcare when needed [30]. The finding was in line with the study in Cote d’Ivoire in which women who were involved in decision-making about their health had 18% higher odds of attending at least four antenatal care visits [31].

Starting antenatal care during the second and third trimesters was associated with a decreased likelihood of completing the continuum of care. Early booking for the antennal care is the entry point for other maternal preventive and curative care services. The findings were in agreement with other previous studies [26]. Initiating antenatal care services within the first trimester gives a woman an opportunity to receive health promotion and preventive care such as screening for proteinuria, early detection and treatment of sexually transmitted infections, immunization against tetanus, nutrition counselling, supplementation of iron and folic acid, prophylactic treatment of malaria and worms [22]. Moreover, early ANC initiation facilitates timely linkage to additional maternal services such as counseling on birth preparedness, HIV testing and treatment, monitoring of maternal conditions like hypertension and anemia, and planning for skilled birth attendance and postnatal care, thereby enhancing overall maternal and neonatal health outcomes. Quality antenatal care reduces maternal mortalities and improves the survival of newborns. The study revealed higher quality of care than the 13% found in Mozambique [32] which could be attributed to different management practices in the two settings.

### Strength and limitations

This is the first study to provide a comprehensive analysis of maternal completion of care in two rural sub-counties of Kenya and the Mwanza region. The findings provide context-specific findings which could aid in co-designing further interventions to improve maternal and newborn health in these regions. The results of the study contributed to the existing knowledge of the role of early antenatal care initiation, women involvement in decision making on their own health in the whole spectrum of the maternal continuum of care.

The study included variables that were collected to respond to the project management framework and might not have considered all the variables explaining the maternal continuum of care such as exposure to media, political goodwill and social-cultural determinants among others. Being a cross-sectional study which could not allow the study of a cause-and-effect relationship between the outcomes and exposure variables was another limitation which limits the the analysis to determine causality. The study also included a few numbers of teenagers which might not be representative of the teenage population in the study settings. Future studies involving youths is therefore recommended.

Finaly, the situation might have changed due to different interventions, government and stakeholder support provided in these regions and the study might not reflect the current situation of maternal continuum of care in these regions. This calls for new studies to provide current information in line with investments in maternal and newborn health and to provide more information on the quality of the services received.

## Conclusion

The study found less than half of the women completed continuum of care during the previous pregnancy which was majorly contributed to low uptake of maternal postnatal care.. The odds of completing the continuum of care increased with the at least secondary level of education and having received high quality antenatal care services and decreased among women who were not involved in decision making about their own health and those who had delayed initiation of antenatal care visits. To effectively increase the uptake of women completing continuum of care, programs targeting female involvement in decision making concerning their own health, early initiation of antenatal care visits and education empowerment among girls should be encouraged in these settings. In addition, more interventions are necessary to strengthen antenatal care which is the entry point for skilled birth attendance and immediate maternal postnatal care after delivery.

## Figures and Tables

**Table 1:** Demographic and reproductive characteristics of women who delivered two years before the end line surveys in regions of Kenyaand Tanzania, 2020 and 2021.

Variable	Overall N = 1,354^1^	Kenya N = 305^1^	Tanzania N = 1,049^1^	p-value^2^
	n (%)	n (%)	n (%)	
**Maternal age (years), (Median (IQR))**	27.0 (23.0–33.0)	26.0 (22.0–32.0)	27.0 (23.0–33.0)	0.057
**Age (years)**				0.028
15–19	95 (7.0)	30 (9.8)	65 (6.2)	
20–34	1,002 (74.0)	228 (74.8)	774 (73.8)	
35–49	257 (19.0)	47 (15.4)	210 (20.0)	
**Marital status**				0.005
Currently married	1,039 (76.7)	250 (82.0)	789 (75.2)	
In a union	106 (7.8)	11 (3.6)	95 (9.1)	
Not in union	209 (15.4)	44 (14.4)	165 (15.7)	
**Level of education**				<0.001
No formal	158 (11.7)	52 (17.0)	106 (10.1)	
Primary	866 (64.0)	162 (53.1)	704 (67.1)	
Secondary and higher	330 (24.4)	91 (29.8)	239 (22.8)	
**Wealth quintile**				<0.001
1	387 (28.6)	169 (55.4)	218 (20.8)	
2	472 (34.9)	106 (34.8)	366 (34.9)	
3	252 (18.6)	10 (3.3)	242 (23.1)	
4	169 (12.5)	7 (2.3)	162 (15.4)	
5	74 (5.5)	13 (4.3)	61 (5.8)	
**Decision-making on respondent’s health**				<0.001
Woman alone	322 (23.8)	86 (28.2)	236 (22.5)	
Woman with partner	446 (32.9)	58 (19.0)	388 (37.0)	
Not involved	586 (43.3)	161 (52.8)	425 (40.5)	
**Initial ANC (Median (IQR))**	16.0 (12.0–20.0)	20.0 (14.0–24.0)	16.0 (12.0–20.0)	<0.001
**Initiation of first ANC**				<0.001
First trimester	546 (40.8)	73 (24.9)	473 (45.3)	
Second trimester	548 (41.0)	132 (45.1)	416 (39.8)	
Third trimester	243 (18.2)	88 (30.0)	155 (14.8)	
**Quality of ANC services**				<0.001
Inadequate	460 (34.0)	58 (19.0)	402 (38.3)	
Adequate/High-quality	894 (66.0)	247 (81.0)	647 (61.7)	
**Wanted of last pregnancy**				0.010
Wanted	716 (52.9)	181 (59.3)	535 (51.0)	
Unwanted	638 (47.1)	124 (40.7)	514 (49.0)	
**ANC visits**				<0.001
0–3	326 (24.3)	96 (32.2)	230 (22.0)	
4+	1,018 (75.7)	202 (67.8)	816 (78.0)	
**Skilled birth attendance**				<0.001
Unskilled	165 (12.2)	61 (20.0)	104 (9.9)	
Skilled	1,189 (87.8)	244 (80.0)	945 (90.1)	
**Postnatal care**				0.110
More than 2 days	354 (37.8)	63 (32.8)	291 (39.1)	
Within 2 days	583 (62.2)	129 (67.2)	454 (60.9)	
**Maternal continuum of care**	421 (31.1)	82 (26.9)	339 (32.3)	0.071

1 Median (IQR) or Frequency (%)

2 Wilcoxon rank sum test; Pearson's Chi-squared test; ANC: Antenatal care; IQR: Interquartile range

**Table 2:** Coverage of different components of maternal health care utilization and CoC among women in regions of Kenya and Tanzania

	Total	Kenya (n (%))	Tanzania (n (%))						
Variable	Overall	Kilifi	Kisii	Buchosa	Ilemela	Magu	Nyamagana	Sengerema	Ukerewe
	N = 1,354	N = 192	N = 113	N = 157	N = 422	N = 153	N = 155	N = 99	N = 63
**ANC visits**									
0–3	326 (24.3)	60 (32.3)	36 (32.1)	35 (22.3)	79 (18.7)	33 (21.9)	44 (28.6)	22 (22.2)	17 (27.0)
4+	1,018 (75.7)	126 (67.7)	76 (67.9)	122 (77.7)	343 (81.3)	118 (78.1)	110 (71.4)	77 (77.8)	46 (73.0)
**Skilled birth attendance**									
Unskilled	165 (12.2)	52 (27.1)	9 (8.0)	5 (3.2)	36 (8.5)	17 (11.1)	17 (11.0)	15 (15.2)	14 (22.2)
Skilled	1,189 (87.8)	140 (72.9)	104 (92.0)	152 (96.8)	386 (91.5)	136 (88.9)	138 (89.0)	84 (84.8)	49 (77.8)
**Postnatal care**									
>2 days	354 (37.8)	33 (30.6)	30 (35.7)	47 (38.2)	110 (35.6)	58 (52.7)	31 (33.0)	40 (58.8)	5 (12.2)
Within 2 days	583 (62.2)	75 (69.4)	54 (64.3)	76 (61.8)	199 (64.4)	52 (47.3)	63 (67.0)	28 (41.2)	36 (87.8)
**Maternal CoC**	421 (31.1)	48 (25.0)	34 (30.1)	63 (40.1)	151 (35.8)	37 (24.2)	45 (29.0)	18 (18.2)	25 (39.7)

CoC: Continuum of care

**Table 3:** Logistic regression analysis of factors associated with the different components of maternal health care utilization and CoC among women in regions of Kenya and Tanzania.

Variable	Dependent variable			
	Model I	Model II	Model III	Model IV
	(ANC->ANC4+)	(ANC4+—>SBA)	(SBA->PNC)	(CoC)
**Age (years)**				
15–19	Ref	Ref	Ref	Ref
20–34	0.66 (0.31–1.23)	0.27**(0.08–0.68)	1.28 (0.74–2.20)	0.80 (0.49–1.34)
35–49	0.82 (0.39–1.68)	0.32**(0.09–0.87)	1.42 (0.76–2.67)	0.91 (0.51–1.62)
**Own health**				
Alone	Ref	Ref	Ref	Ref
Joint with the partner	1.16 (0.72–1.85)	1.92**(1.13–3.27)	1.11 (0.75–1.65)	1.28 (0.91–1.81)
Not involved	0.82 (0.53–1.25)	0.85 (0.55–1.31)	0.65**(0.12–0.94)	0.66**(0.47–0.92)
**Marital status**				
Currently married	Ref	Ref	Ref	Ref
In union	0.72 (0.41–1.31)	0.74 (0.42–1.40)	0.92 (0.52–1.64)	0.74 (0.45–1.17)
Not-in union	1.39 (0.86–2.29)	0.88 (0.54–1.46)	0.90 (0.58–1.39)	0.88 (0.60–1.29)
**Level of education**				
No formal	Ref			
Primary	1.03 (0.62–1.69)	1.64**(1.02–2.57)	1.11 (0.70–1.76)	1.06 (0.71–1.61)
Secondary and higher	2.01**(1.09–3.68)	2.89**(1.56–5.47)	1.45 (0.85–2.45)	1.78**(1.13–2.84)
**Wealth quintile**				
1	Ref	Ref	Ref	Ref
2	1.18 (0.80–1.75)	0.95 (0.63–1.43)	0.90 (0.63–1.29)	0.94 (0.69–1.29)
3	1.34 (0.83–2.20)	1.21 (0.72–2.09)	0.79 (0.52–1.19)	0.85 (0.59–1.22)
4	1.29 (0.73–2.33)	1.38 (0.73–2.75)	0.76 (0.47–1.24)	0.76 (0.50–1.15)
5	0.73 (0.35–1.57)	4.03*(1.18–25.27)	0.55**(0.30–0.997)	0.59*(0.32–1.05)
**Initiation of ANC**				
First trimester	Ref	Ref	Ref	Ref
Second trimester	0.19***(0.12–0.30)	0.61**(0.41–0.91)	0.96 (0.71–1.30)	0.63***(0.49–0.81)
Third trimester	0.02***(0.40–0.77)	0.58**(0.36–0.95)	1.07 (0.71–1.61)	0.13***(0.08–0.21)
**Previous pregnancy wanted**				
Wanted	Ref	Ref	Ref	Ref
Unwanted	0.56***(0.40–0.77)	1.07 (0.75–1.53)	0.94 (0.71–1.26)	0.78*(0.61–1.01)
**Quality of ANC**				
Inadequate	Ref	Ref	Ref	Ref
Adequate/High-quality	0.92 (0.65–1.31)	1.56**(1.08–2.23)	1.33*(0.97–1.81)	1.50***(1.14–1.97)
Observations	1337	1337	884	1337
AIC	1017.70	928.11	1187.28	1544.83

Note:

* p<0.1

** p<0.05

*** p<0.01; **CoC**: Continuum of care; **ANC**: Antenatal care; **AIC**: Akaike Information Criterion; **SBA**: Skilled birth attendance; **PNC**: Postnatal care

## Data Availability

The datasets generated and analyzed for this study are available on request from Prof. Marleen Temmerman through the email address: marleen.termmerman@aku.edu. and based on an official data transfer and user agreement sent to the director of the Centre of Excellence in Women and Child Health East Africa at the Aga-Khan University.

## Abbreviations

AIC: Akaike Information Criterion
ANC: Antenatal care
AQCESS: Access to Quality Care through Extending and Strengthening Health Systems
CoC: Continuum of care
IMPACT: Improving Access to Reproductive, Maternal and Newborn Health in Mwanza
IQR: Interquartile range
MMR: Maternal mortality ratio
PNC: Postnatal care
SBA: Skilled birth attendance
SDGs: Sustainable Development Goals (SDGs)
WHO: World Health Organization
